# Evaluation of five commercial DNA extraction kits using *Salmonella* as a model for implementation of rapid Nanopore sequencing in routine diagnostic laboratories

**DOI:** 10.1099/acmi.0.000468.v3

**Published:** 2023-02-21

**Authors:** Shannon H. C. Eagle, James Robertson, D. Patrick Bastedo, Kira Liu, John H. E. Nash

**Affiliations:** ^1^​ National Microbiology Laboratory, Public Health Agency of Canada, Guelph, Ontario, Canada; ^2^​ National Microbiology Laboratory, Public Health Agency of Canada, Toronto, Ontario, Canada; ^3^​ Patented Medicine Prices Review Board, Ottawa, Ontario, Canada

**Keywords:** Oxford Nanopore sequencing, DNA extractions, public health, bacterial genomics, whole-genome sequencing, long-read sequencing

## Abstract

Oxford Nanopore long-read sequencing offers advantages over Illumina short reads for the identification and characterization of bacterial pathogens for outbreak detection and surveillance activities within a diagnostic public health laboratory context. Compared to Illumina, Nanopore is more cost-effective for small batches, has a lower capital cost and has a faster turnaround time, in addition to the ability to assemble complete bacterial genomes. The quantity and quality of DNA required for Nanopore sequencing are greater than for Illumina, and the DNA extraction methods recommended for obtaining high-molecular-weight DNA are different from those typically used in diagnostic laboratories. Using a *Salmonella* isolate with a previously closed PacBio genome as a model Enterobacteriaceae organism, we evaluated the quantity, quality and fragmentation of five commercial DNA extraction kits. Nanopore sequencing performance was evaluated for the top three methods: Qiagen EZ1 DNA Tissue, Qiagen DNeasy Blood and Tissue, and a modified, in-house version of the MasterPure Complete DNA and RNA purification. To evaluate the effect of post-extraction DNA purification methods, we subjected extracted DNA from the three selected extraction methods to purification by AMPure beads or ethanol precipitation and compared these outputs with untreated DNA as a control. All methods are suitable for routine whole-genome sequencing (WGS), since all 60 replicates had very high genome recovery rates, with ≥98 % of the reference genome covered by mapped Nanopore reads. For 85 % of the replicates, assembly was able to produce a complete, circular chromosome using either Flye or Canu. In most cases, it is recommended to move directly from extraction to sequencing, as untreated DNA had the highest rates of genome closure regardless of extraction method. Using our evaluation criteria, the Qiagen DNeasy Blood and Tissue kit was found to be the best overall method due to its low cost, ability to scale from single tubes to 96-well plates, and high consistency in yield and sequencing performance.

## Impact statement

This study provides useful guidance to diagnostic laboratories looking to implement Nanopore sequencing on suitable DNA extraction and purification methods. We evaluated five commercial DNA extraction kits for use with Nanopore sequencing in diagnostic public health laboratories. We used a previously closed *Salmonella* genome as a model organism for Enterobacteriaceae since it is one of the most commonly sequenced bacterial pathogens for public health. Extraction kits were rated based on technical performance in addition to labour and consumable costs, and scalability. DNA purification methods are frequently employed to concentrate and change buffers of extracted DNA. We evaluated the effects of AMpure bead and ethanol-based purifications of DNA and found that untreated DNA performed the best. The three most promising extraction methods were further evaluated based on sequencing results, including the ability to recover the chromosome of the reference and complete assembly of two plasmids. All three of the DNA extraction methods had very high rates of genome recovery and are suitable for generation of Whole Genome Sequencing (WGS) for bacterial isolates. We demonstrate that laboratories have flexibility in the selection of their DNA extraction method for Nanopore sequencing and are not limited to high-molecular-weight extraction methods.

## Data Summary

Raw sequence data for all replicates have been deposited in National Center for Biotechnology Information (NCBI) BioProject PRJNA768992 (accession number SRR16525216 to SRR16525275). Three supplementary files are available with the online version of this article.

The authors confirm all supporting data, code and protocols have been provided within the article or through supplementary data files.

## Introduction

Many public health reference and diagnostic laboratories use whole-genome sequencing (WGS) for pathogen identification and characterization, in addition to surveillance and outbreak detection activities. WGS is a multi-step process consisting of DNA extraction, sequencing library preparation and post-sequencing analysis. There are numerous DNA extraction protocols and commercial kits available, and selecting the optimal method for a given laboratory context is complex. There are numerous factors to consider when implementing extraction methods for WGS, including: hands-on time, overall turnaround time, consumable cost, reproducibility, throughput and ease of use. Automated methods can offer the benefits of ease of use, reproducibility and reduced hands-on time, in addition to the potential for higher throughputs. For public health reference and diagnostic laboratories, it may be advantageous for an extraction method to have multiple formats that scale from small to large sample volumes, depending on shifting needs. In the case of surveillance samples, where turnaround time is not critical, samples can be batched in plate format to gain efficiencies in time and cost, but where turnaround time is critical, the low throughput format may be preferable. Batch size is also an important consideration during sequencing technology selection. Illumina sequencing (e.g. MiSeq) requires isolates to be batched together to make it cost-effective, whereas the MinION from Oxford Nanopore Technologies (ONT) is cost-effective for smaller batches or even for single samples due to the ability of ONT’s flow cells to be washed and reused until there are no viable pores remaining. Further, data from ONT sequencing can be analysed in real time, meaning the run can be stopped as soon as sufficient data have been obtained, offering quick diagnoses.

ONT sequencing requires both a high quality and quantity of DNA and so phenol : chloroform is one of the more popular methods for extraction of pure, high molecular weight (HMW) used in the ONT community. These extractions are time-consuming, involve toxic reagents and need to be performed in a containment hood. There are many commercial DNA extraction kits on the market that do not require the use of a chemical containment hood, are highly reproducible and are less time-consuming than phenol : chloroform. These kits utilize a variety of mechanisms, such as silica columns, salt precipitation, or magnetic particles, which each have their own benefits and drawbacks for producing HMW DNA. Compared to phenol : chloroform, silica column-based methods are more likely to generate fragmented DNA due to increased DNA shearing that occurs when the DNA passes through the silica membrane. For this method, following cell lysis, DNA binds to a silica membrane in a column, the DNA is washed while bound to the membrane, and finally, the DNA is eluted. Salt precipitation-based methods are less likely to cause DNA fragmentation compared to silica column-based methods due to the fact that DNA does not pass through a membrane. These methods use a desalting procedure, which causes the cell debris to precipitate out of solution, leaving the DNA in the supernatant; the DNA is subsequently precipitated out of solution using isopropanol and washed using ethanol; and finally, the DNA is resuspended. Similar to salt precipitation-based methods, magnetic particle-based methods are gentle on DNA. In this type of method, the DNA binds to the magnetic particles; the DNA is washed while bound to the magnetic particles; and finally, the DNA is eluted from the magnetic particles. The efficiency of the reagents and the number of washing steps can impact on yield and purity.

Although further post-extraction purification of DNA is not essential and has the potential to cause additional shearing due to additional manipulation, it can be useful for removal of contaminants, exchange of the elution buffer and/or concentration of the DNA. Buffer exchange allows for the removal of enzymatic inhibitors present in the elution buffer that may interfere with downstream library preparation (e.g. EDTA can interfere with enzymatic activity). Examples of purification method mechanisms include ethanol precipitation and magnetic beads. Ethanol precipitation is based on the principle that DNA will precipitate out of solution when mixed with salt and alcohol, allowing contaminants that remain soluble to be washed away. Following precipitation, the DNA is spun to create a pellet; the supernatant is removed; the DNA pellet is washed; and the DNA pellet is resuspended in a buffer. Magnetic bead purification uses solid phase reversible immobilization (SPRI) technology. This purification method is similar to the magnetic particle extraction method post-lysis. Automation and high throughput is feasible for the magnetic bead purifications, but not for the ethanol precipitation.

WGS is currently dominated by short read (<300 bp) technologies such as those developed by Illumina. Illumina offers high-quality reads, low-input DNA requirements (1–500 ng), and minimal requirements on DNA fragment length. Further, since Illumina is a mature technology, there are many supports and tools available. In contrast, the more recently developed long-read technology by ONT, in theory, offers read lengths the length of the nucleic acid molecule being sequenced. The longest ONT read to date is 4.2 Mb [1]. ONT read length depends on the actual length of the DNA fragments used in library preparation; therefore, using higher molecular weight DNA for library preparation will allow for the generation of longer reads. Further, ONT can use amplification-free library preparations, but this requires a large quantity of contaminant-free DNA. ONT offers two amplification-free library preparation kits: the Ligation Sequencing kit and the Rapid Sequencing kit. The Rapid kit can be completed in 10 min and requires only 400 ng of input DNA, whereas the Ligation kit takes an hour, needs 1000 ng of DNA, and needs other reagents not supplied with the kit.

Long reads, such as those generated in ONT sequencing, facilitate the assembly of complete, circular bacterial genomes because they span and can therefore resolve repeat regions [2]. In bacteria, the longest repetitive region within the genome is usually the 7 kb rRNA operon [2]. Many of the routine analyses performed using WGS data in public health laboratories do not require complete, circular genomes, but in certain cases it may be necessary to generate an internal, high-quality reference, for example when performing single-nucleotide polymorphism (SNP) analysis of outbreak strains. Many complete, circular reference genomes exist, but Valiente-Mullor *et al.* [3] demonstrated that the choice of reference genome can significantly alter the number of SNPs detected and the subsequent phylogenetic tree generated, and so it is beneficial to generate a high-quality reference genome to maximize the detection of SNPs. Localization of antimicrobial resistance (AMR) genes, on the other hand, requires a complete genome in order to be fully confident when determining the context and copy number for each gene. Multiple algorithms have been developed to identify plasmids using short reads [4–6], but the results can be confounded by repetitive DNA. For example, Sheppard *et al.* [7] demonstrated that long reads were required to determine the genomic location of a carbapenem resistance element. By determining its genomic location, they were able to show that its spread was due to both horizontal gene transfer and homologous recombination [7]. Genomic location is important because genes present in mobile genetic elements (i.e. plasmids, transposons and bacteriophages) can spread within and between bacterial populations. Therefore, in microbiology long reads are necessary for applications that require certainty in the genetic context of a given element, such as the monitoring of AMR gene dissemination.

Genome assembly is a complex process requiring a variety of different steps to produce high-quality bacterial genomes. Long reads produced by ONT have a higher error rate than Illumina reads and necessitate a different genome assembly approach from the established short read assemblers such as SPAdes [8]. There have been efforts to characterize newly developed long read assemblers and to determine which ones produce the most contiguous assemblies with the lowest error rates [9]. In one study that evaluated the performance of eight long read assemblers, no single assembler was best for all datasets across all metrics, but Flye [10] and Miniasm [11] were found to perform well for producing complete, circular genomes [9]. Canu [12] was found to produce high-quality assemblies but did not circularize genomes well [9]. In addition to assemblers designed specifically for assembling genomes exclusively from long reads, the sequencing data from both short and long read technologies can be combined into a hybrid assembly [13, 14]. A hybrid assembly can provide a more polished complete reference genome, due to the fact that short reads tend to have higher quality than long reads [15]. Unicycler is a common hybrid assembler for bacterial genomes that is also capable of short read-only and long read-only assembly [16]. Unicycler uses SPAdes [8] for lllumina short read assembly and Miniasm+Racon [11, 17] for long read assembly.

ONT long read sequencing offers many benefits to reference and diagnostic laboratories, including: read length, low capital cost and quick turnaround times; however, implementation of ONT sequencing requires the testing and validation of several processes, including DNA extraction. Our goal is to evaluate commonly used DNA extraction kits and extraction purification methods for ONT sequencing in the context of their potential use in routine diagnostic laboratories. We chose to use *Salmonella enterica *, a Gram-negative bacteria, as it is a major cause of foodborne disease [18], it has been listed as a serious threat in terms of AMR [19], and the results should be applicable to other members of Enterobacteriaceae.

## Methods

**Fig. 1.**
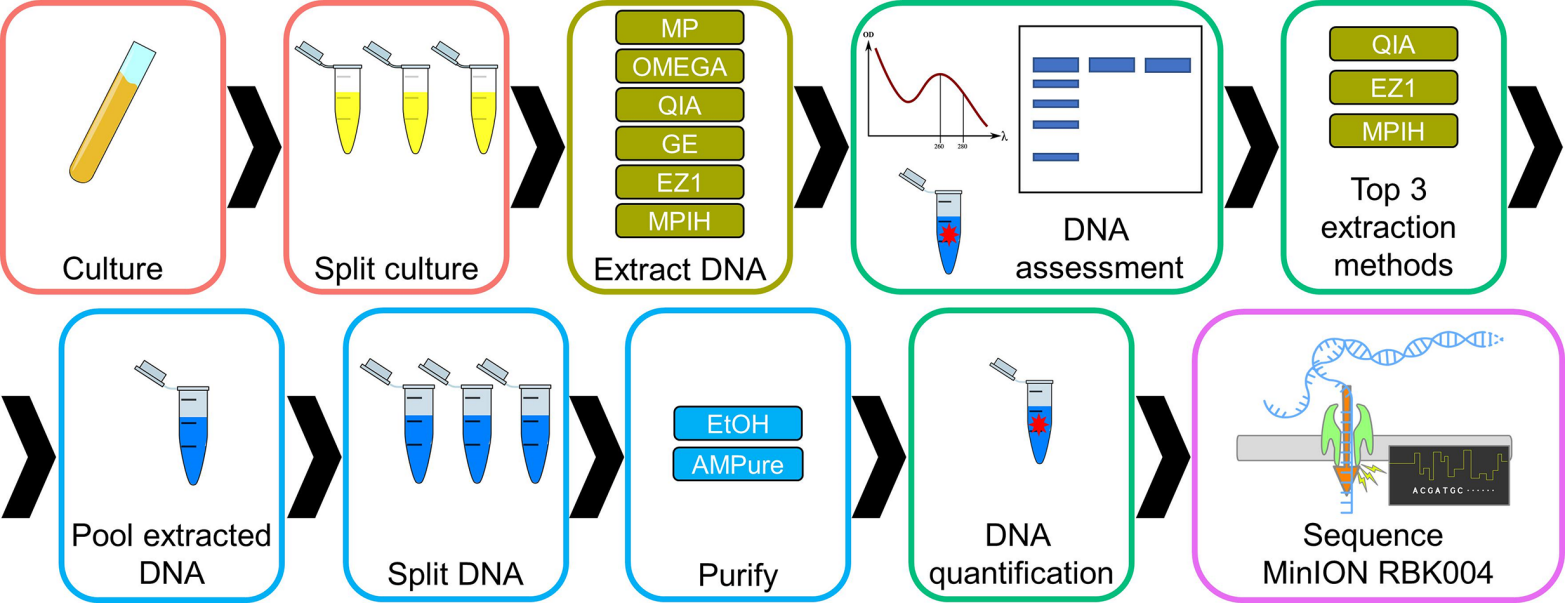
Flow chart overview of the laboratory methods. The extraction methods are: Lucigen MasterPure Complete DNA and RNA Purification kit (MP), Omega EZNA Bacterial DNA kit (OMEGA), Qiagen DNeasy Blood and Tissue (QIA), GE Illustra Bacteria genomicPrep Mini Spin kit (GE), Qiagen EZ1 DNA Tissue kit (EZ1) and an in-house variation of Lucigen MasterPure Complete DNA and RNA Purification kit (MPIH) developed at the National Microbiology Laboratory. The purification methods are: ethanol precipitation (EtOH) and Beckman Coulter AMPure XP Beads (AMPure).

A flow chart of the laboratory methods is shown in Fig. 1.

### Culturing conditions

*S. enterica* subsp. *enterica* ser. Heidelberg strain SA02DT10168701 (CP012921.1) contains a 37 kb IncX1 plasmid (CP012923.1) and a 100 kb IncI1 plasmid (CP012922.1); it was selected for this study because it was sequenced previously with Illumina and PacBio and a complete, circular genome was obtained, which provided a high-quality reference suitable for comparative analyses [20]. Cultures were grown aerobically to saturation in Luria-Bertani (LB) broth overnight at 37 °C (Fig. 1).

### Extraction

Six different extraction methods were tested using five different commercial extraction kits representing salt precipitation-, silica column- and magnetic particle-based methods (Table 1): Lucigen MasterPure Complete DNA and RNA Purification kit, Omega EZNA Bacterial DNA kit (OMEGA), Qiagen DNeasy Blood and Tissue (QIA), GE Illustra Bacteria genomicPrep Mini Spin kit (GE) and Qiagen EZ1 DNA Tissue kit (EZ1). Two different protocols were used for the MasterPure kit: the manufacturer’s protocol for total nucleic acid purification (MP) and a modified, in-house method (MPIH) developed at the National Microbiology Laboratory (Public Health Agency of Canada). Extractions were performed in triplicate, starting with either 0.5 or 1 ml of culture based on manufacturers’ recommendations (Fig. 1, Table 1). Manufacturers’ protocols with modifications are described in File S1 (available in the online version of this article). DNA was eluted in 100 µl of molecular-grade water, with the exception of the DNA extracted with EZ1, which was eluted in 100 µl of elution buffer.

**Table 1.** Summary of extraction methods. The kit cost is the cost of the kit per reaction (rxn), whereas the total cost is the cost of one reaction, including kit cost, laboratory reagents and consumables. The kit cost for MPIH includes the cost of a few additional reagents due to increased amounts of these reagents being used compared to the manufacturer’s protocol

Code	Full kit name and company	Method type	Amount of culture (ml)	Kit cost (CAD, per rxn)	Total cost (CAD, per rxn)	Total time (12 rxn)	Hands-on time (12 rxn)
**MP**	MasterPure Complete DNA and RNA Purification kit (Lucigen)	Salt precipitation	0.5	$4.19	$6.56	1 h 55 m	1 h 25 m
**OMEGA**	E.Z.N.A. Bacterial DNA Kit (OMEGA)	Silica column	1.0	$7.33	$10.32	2 h 20 m	1 h 20 m
**QIA**	DNeasy Blood and Tissue Kit (Qiagen)	Silica column	0.5	$3.54	$5.99	2h	1h
**GE**	Illustra Bacteria genomicPrep Mini Spin Kit (GE)	Silica column	1.0	$5.69	$7.86	1 h 10 m	1 h 10 m
**EZ1**	EZ1 DNA Tissue Kit (Qiagen)	Magnetic particles	1.0	$11.25	$12.32	3 h 35 m	35 m
**MPIH**	Modified, in house MasterPure Kit	Salt precipitation	0.5	$5.34	$8.12	1 h 55 m	1 h 40 m

The cost of the kit per reaction and total cost per reaction were calculated using pre-tax totals in CAD as of June 2020. The cost of the kit for MPIH includes the cost of a few additional reagents from MasterPure due to the increased volume used of these reagents in the in-house method compared to the manufacturer’s protocol. The total cost per reaction includes the cost of the kit as well as the cost of any additional laboratory reagents and consumables required to perform the extraction (e.g. tubes, tips, ethanol). Equipment requirements were not factored into the cost of the kit. Both MPIH and EZ1 require equipment that may not be found in all laboratories: a chilled centrifuge and EZ1 robots, respectively. The total time and hands-on time were determined for a batch of 12 reactions. Hands-on time excludes all steps with incubation periods of 15 min or greater.

### DNA assessment

Samples were checked for quantity, purity, and fragment length after extraction (Fig. 1). Quantification was done using the Qubit Broad Range Quantification Kit (Invitrogen) as per manufacturer’s protocols. The Nanodrop 8000 was used to assess sample purity (A260/280 and A260/230 ratios). Finally, fragment length was assessed by electrophoresis using a 0.5 % agarose gel in TBE buffer, stained with GelRed Nucleic Acid Gel Stain (Biotium), and visualized under UV light with a BioRad GelDoc XR +and ImageLab (v. 2.5.1). Gels were analysed using ImageJ [21] and the total area of each gel lane was calculated. The DNA ladder was used to select the area corresponding to ≥20 kb. Fragments ≥20 kb were considered high molecular weight DNA because fragments of this size would have a high probability of producing a fragment longer than 7 kb (the size of the rRNA operon in most bacterial genomes) during library preparation with ONT’s Rapid Sequencing kit. Since the amount of input DNA varies between samples the DNA fragment quality is expressed as a percentage of DNA which is above 20 kb for each individual sample.

Extraction methods were ranked based on the following factors: quantity, variability, distance from optimal purity, fragments≥20 kb, total cost, hands-on time, and total time. Factors were scaled to between 0.00 and 1.00 by dividing the difference from the minimum value by the range between maximum and minimum values; therefore, the method with the highest value received a score of 1.00.

$$y_{i}=\frac{x_{i}-x_{min}}{x_{max}-x_{min}}$$

However, in the case of almost all factors (quantity and fragments≥20 kb being the exceptions), a high value indicates a worse method, e.g. a score of 1.00 for variability meant that the method had the highest variability. Therefore for those factors, to ensure that when the values were summed the best method would be the one with the highest sum, the normalized value was subtracted from 1.

### Purification

Two purification methods were used (Fig. 1): ethanol precipitation (EtOH) and Beckman Coulter AMPure XP Beads (AMPure).

The EtOH method was based on several laboratory protocols [22]: one-tenth the volume of 3M Sodium acetate, pH 5.2, was added and mixed gently by flicking, followed by 2 to 3 volumes of ice-cold 95–100 % ethanol. Samples were incubated at −20 °C for 1 h. After incubation, samples were centrifuged for 30 min at 14,000 x *g*. Following the spin, the supernatant was removed. Samples were washed by adding 500 µl of 70 % ethanol, spinning for 15 min at 14,000 x *g*, and removing the supernatant. Any remaining ethanol was allowed to evaporate for up to 20 min before resuspension of the pellet in 20 µl of molecular grade water and mixing by pipette.

The AMPure method followed the manufacturer’s protocol with a few modifications, the amount of beads used was 1X vs. 1.8X the volume of the sample, samples were flicked to mix vs. pipetting, the optional air-drying step was performed for up to 20 min to remove remaining ethanol, and DNA was eluted in 20 µl of molecular grade water vs. 40 µl of elution buffer.

### Sequencing

Five sequencing runs were performed with 12 replicates per run for a total of 60 replicates. The 60 replicates were split across nine treatments. Treatments consist of a specific combination of extraction and purification method and are referred to in text in the following format Extraction-Purification. The nine treatments are: EZ1-AMPure (*n*=9), EZ1-EtOH (*n*=9), EZ1-Unpurified (*n*=3), MPIH-AMPure (*n*=9), MPIH-EtOH (*n*=9), MPIH-Unpurified (*n*=9), QIA-AMPure (*n*=4), QIA-EtOH (*n*=4), and QIA-Unpurified (*n*=4).

Libraries were prepared with ONT’s Rapid Barcoding Kit (SQK-RBK004) according to the manufacturer’s protocol (Fig. 1). For most replicates the recommended 400 ng of DNA was used; however, there were a few exceptions. Library preparation reactions using QIA-Unpurified (*n*=4) and EZ1-Unpurified (*n*=3) as template used 196 and 125 ng of DNA, respectively. For QIA, the DNA concentration was insufficient to obtain 400 ng within the specified volume (7.5 µl). For EZ1, the DNA needed to be diluted (2 µl in 5.5 µl) as volumes greater than 2 µl have been shown to cause inhibition during library preparation, likely due to the sodium azide present in the EZ1 elution buffer (data not shown). FLO-MIN106D version 9.4.1 flow cells were used. Flow cells were run for 48 h on a MinION using MinKNOW v. 18.12.6 or 18.12.9 with base-calling on.

### Sequence data quality assessment and circularity checks

Accession numbers for the raw reads are in File S2. Fastq files from each sequencing run were concatenated prior to demultiplexing with qcat (v. 1.0.7) (https://github.com/nanoporetech/qcat). Nanostat (v. 1.1.2) [23] was used to collect the following information for each replicate: mean read length, mean read quality, number of reads, read length N50, total sequenced bases, and longest read. Coverage was calculated by dividing the total sequenced bases by the genome size of the reference (4,888,155 bp).

For each sample, assemblies were prepared using a variety of assemblers and assembly parameters for a total of six different assemblies per replicate: Canu, Flye, Unicycler_hybrid, Unicycler_hybrid_bold, Unicycler_canu, and Unicycler_flye. Long-read assemblies were generated using both Canu (v. 1.8) [12] and Flye (v. 2.6) [10]. Several replicates had coverage less than 10×; by default Canu will not assemble a sample if the coverage is less than 10×. However, by lowering the stopOnLowCoverage value Canu would proceed with assembly as long as the coverage was greater than the stopOnLowCoverage value. Flye was run with two polishing iterations (-i) and was set to rescue unassembled plasmids (--plasmids). Unicycler (v. 0.4.4) [16] was used to generate hybrid assemblies. Hybrid assemblies used both long-reads from ONT and short-reads from Illumina MiSeq v. 3 which were generated previously for the complete, circular genome (SRR2598330). Unicycler was run using four different parameter sets: hybrid with default parameters; hybrid_bold used the bold bridging mode which gives the longest contigs (--mode bold); Unicycler_canu supplied the existing long-read assembly from Canu for that replicate (--existing_long_read_assembly); and Unicycler_flye supplied the existing long-read assembly from Flye for that replicate. Microsoft Excel, R Studio, and Microsoft PowerPoint were used for data analysis and figure generation.

The recovery of the reference genome was characterized in each of the different read-sets by mapping the reads against the reference genome (CP012921.1- CP012923.1) using Miniasm with the default parameters [11]. The mapped reads were sorted and filtered using samtools (v. 1.10) [24]. The number of bases with 0 read coverage was determined using bedtools (v. 2.29.2) [25].

## Results and Discussion

### Extraction

**Fig. 2.**
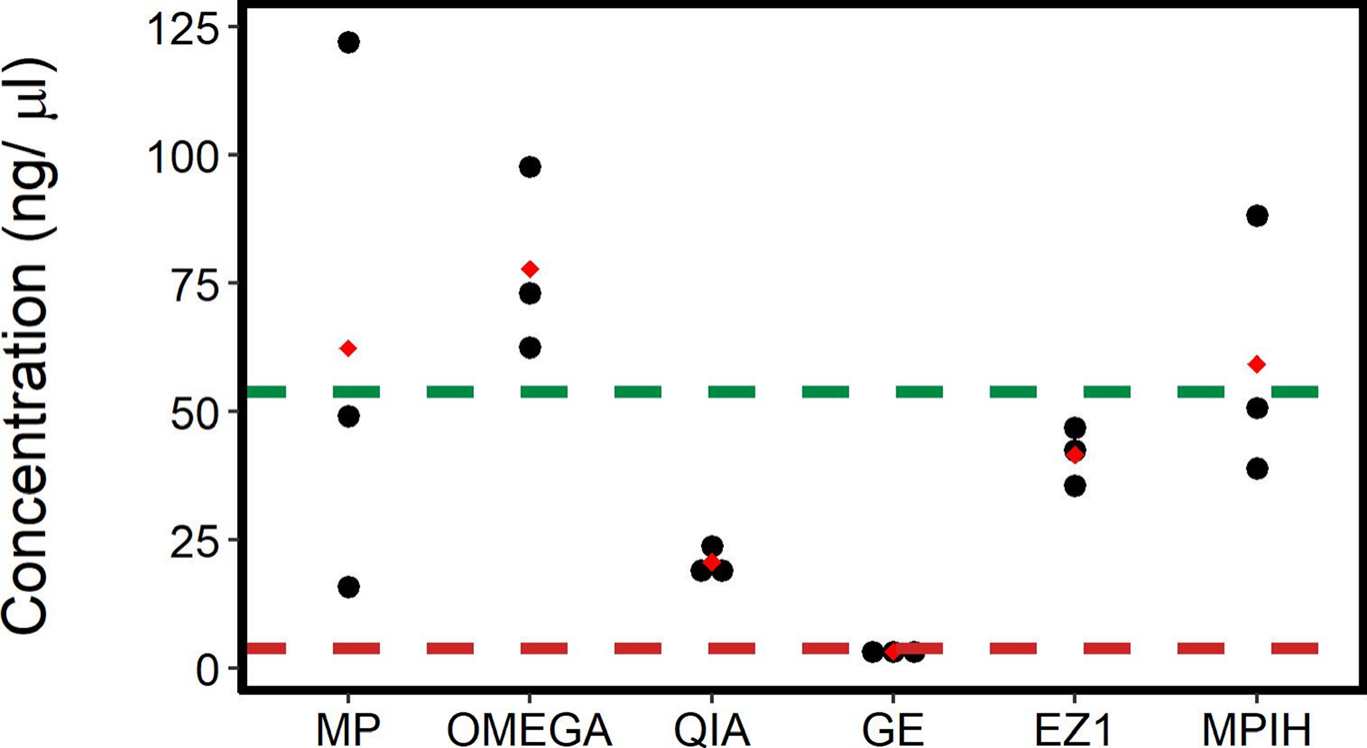
DNA quantification results for the six extraction methods. Three extraction replicates were done for each of the following methods: Lucigen MasterPure Complete DNA and RNA Purification Kit (MP), Omega E.Z.N.A. Bacterial DNA Kit (OMEGA), Qiagen DNeasy Blood and Tissue (QIA), GE Illustra Bacteria genomicPrep Mini Spin Kit (GE), Qiagen EZ1 DNA Tissue Kit (EZ1), and an in-house variation of Lucigen MasterPure Complete DNA and RNA Purification Kit (MPIH) developed at the National Microbiology Laboratory. The red diamond represents the mean for each extraction method. The dotted lines represent the DNA recommendations by Oxford Nanopore Technologies for sufficiently concentrated DNA for sequencing (green, 400 ng in 7.5 µl=53.3 ng/µl) and sufficient quantities (red, the 100 µl extracts must have a concentration of at 4 ng/µl to yield a total of 400 ng).

ONT requires 400 ng of DNA in 7.5 µl for the Rapid Sequencing kit, i.e. at least 54 ng/µl [26]. OMEGA produced extracts with the highest mean DNA concentration (77.8 ng/µl, SD=18.7) and each replicate yielded DNA that was concentrated enough for ONT sequencing (Fig. 2). MP also had a high mean DNA concentration (62.4 ng/µl, SD=74.4), but there was high variability between replicates (Fig. 2). MPIH generated similar mean amounts of DNA (59.3 ng/µl, SD=25.7) compared to the unmodified MP, but with much lower variability between replicates (Fig. 2). The increased amount of several reagents used in MPIH compared to MP might have improved the digestion of cellular matter leading to more consistent DNA yields. The high average DNA concentration for both of the MasterPure methods obscures the fact that just a single replicate for each method exceeds the minimum threshold of 54 ng/µl for direct ONT sequencing, so post-extraction concentration may be necessary (Fig. 2). Both Qiagen methods, EZ1 (41.7 ng/µl, SD=5.3) and QIA (20.7 ng/µl, SD=2.9), had highly consistent yields between replicates, and while the concentration was below the ONT requirement, they both provided sufficient amounts of DNA for ONT sequencing with subsequent concentration (Fig. 2). GE generated an unusably low amount and concentration of DNA with a mean of 3.3 ng/µl (SD=0.3) and a total yield of only 327 ng (Fig. 2). Five of the six methods yielded sufficient quantities of DNA for ONT sequencing (Fig. 2). The three methods with the highest mean concentration (OMEGA, MP, and MPIH) had high variability. Although OMEGA is superior in terms of concentration, both Qiagen methods, EZ1 and QIA, are superior when considering both quantity and variability (Table 2).

**Table 2.** Evaluation of extraction methods using scaled values for each factor. the extraction methods are: Lucigen MasterPure complete DNA and RNA purification kit (MP), Omega E.Z.N.A. Bacterial DNA Kit (OMEGA), Qiagen DNeasy Blood and Tissue kit (QIA), GE Illustra Bacteria genomicPrep Mini Spin Kit (GE), Qiagen EZ1 DNA Tissue Kit (EZ1), and an in-house variation of Lucigen MasterPure Complete DNA and RNA Purification Kit (MPIH) developed at the National Microbiology Laboratory

Code	Method type	Quantity	1−variability	1−distance from optimal purity		Fragments >20 kb	1−total cost	1−hands-on time	1−total time	Score	Rank
				A260/280	A260/230						
**MP**	Salt precipitation	0.79	0.00	0.00	0.90	0.64	0.91	0.23	0.69	4.17	**4**
**OMEGA**	Silica column	1.00	0.67	0.05	1.00	0.68	0.32	0.31	0.52	4.54	**3**
**QIA**	Silica column	0.23	0.95	0.24	0.58	0.90	1.00	0.62	0.66	5.16	**1**
**GE**	Silica column	0.00	1.00	0.00	0.00	0.00	0.70	0.46	1.00	3.17	**5**
**EZ1**	Magnetic particles	0.52	0.90	1.00	0.30	0.94	0.00	1.00	0.00	4.66	**2**
**MPIH**	Salt precipitation	0.75	0.53	0.52	1.00	1.00	0.66	0.00	0.69	5.16	**1**

**Fig. 3.**
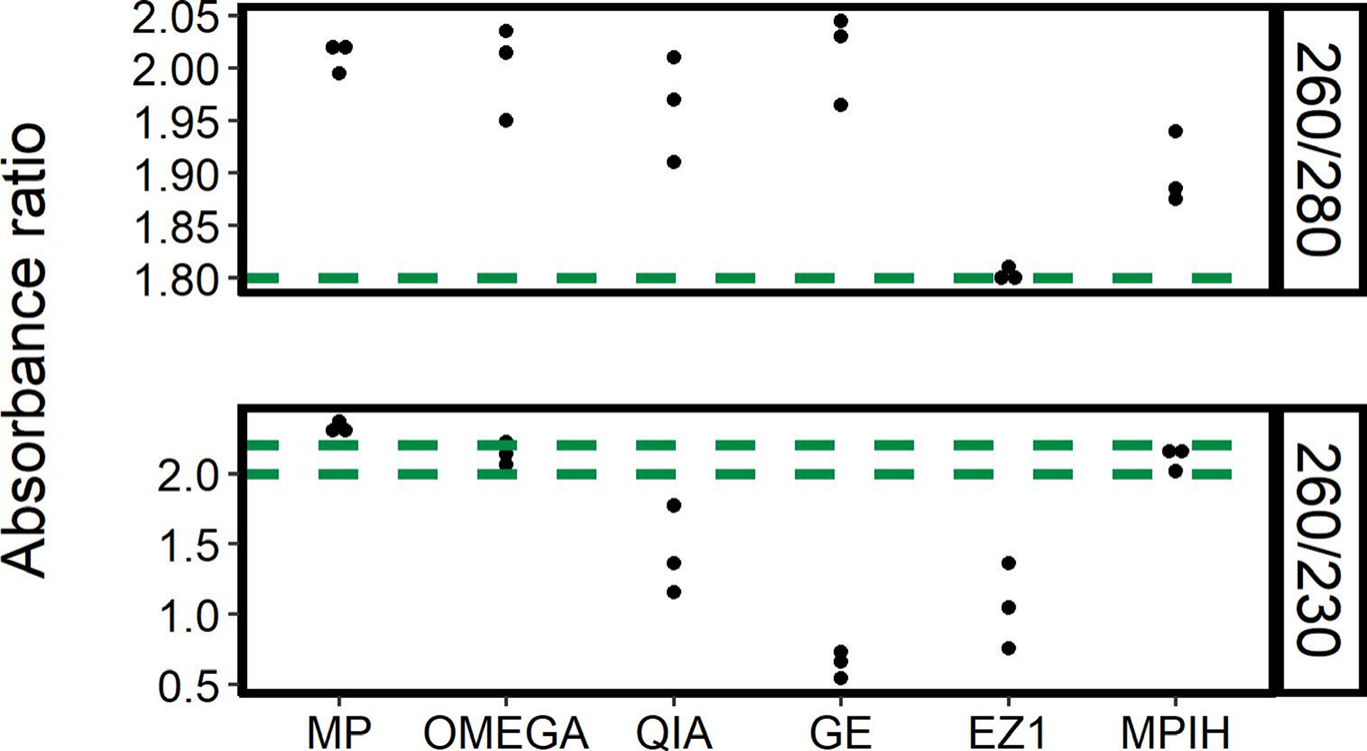
Purity results for the six extraction methods. Nanodrop A260/280 and A260/230 absorbance ratios were used to assess DNA purity. Three extraction replicates were performed for each of the following six extraction methods: Lucigen MasterPure Complete DNA and RNA Purification kit (MP), Omega EZNA Bacterial DNA kit (OMEGA), Qiagen DNeasy Blood and Tissue (QIA), GE Illustra Bacteria genomicPrep Mini Spin kit (GE), Qiagen EZ1 DNA Tissue kit (EZ1), and an in-house variation of Lucigen MasterPure Complete DNA and RNA Purification kit (MPIH) developed at the National Microbiology Laboratory. The green horizontal lines indicate the recommended ratio from Oxford Nanopore Technologies (1.8 for the A260/280 ratio and 2.0 to 2.2 for the A260/230 ratio).

ONT recommends using DNA with an A260/280 ratio of ~1.8 and an A260/230 ratio of 2.0–2.2 [27]. Of the tested methods, MPIH was the closest to the acceptable range for both ratios, with an A260/280 near 1.90 and an A260/230 within the recommended range (Fig. 3). Compared to the modified protocol, the MP protocol had a higher A260/280 ratio, which is indicative of RNA contamination (Fig. 3). The decrease in RNA contamination in MPIH over MP replicates suggests that increasing the amount of RNase had a positive impact on eliminating RNA in the sample. EZ1 consistently gave A260/280 ratios near 1.8, but its A260/230 ratios were well below the recommended value (Fig. 3). From testing in our laboratory the sodium azide present in EZ1’s elution buffer has been shown to lower the A260/230 ratio (data not shown). OMEGA was within the acceptable range for A260/230, suggesting a lack of non-nucleic acid contaminants, which may be due to the fact that this method includes three washes while bound to the column compared to only two washes in the GE and QIA protocols (Fig. 3). Three methods, QIA, GE and MP, were outside the recommended range for both A260/280 and A260/230 ratios (Fig. 3). Since none of the tested extraction methods had both an A260/280 and A260/230 ratio within the ONT’s recommended range, post-extraction purification may be necessary for all of these methods to obtain DNA with sufficient purity for ONT sequencing.

**Fig. 4.**
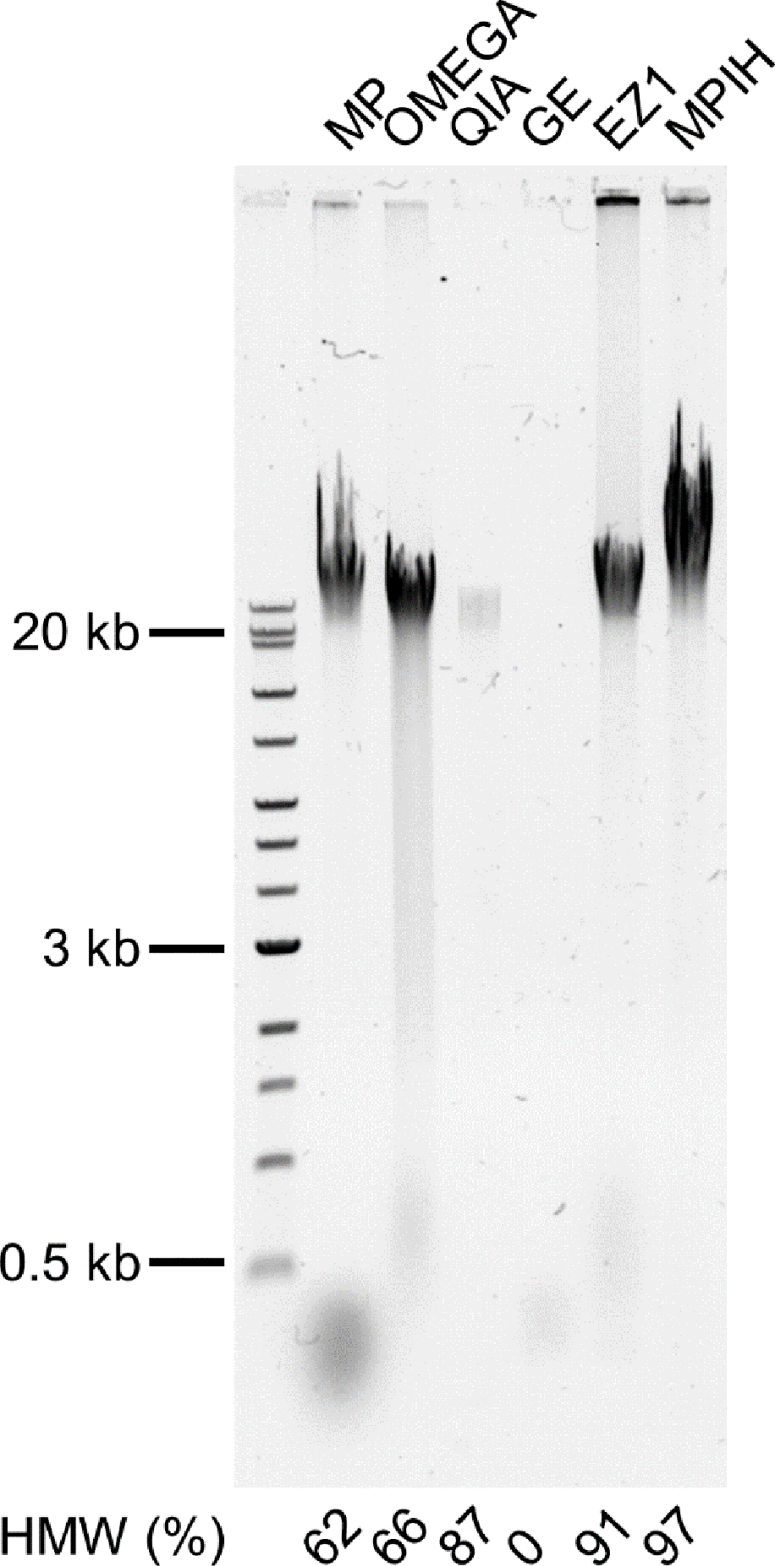
DNA fragment length results for the six extraction methods with 0.5 % agarose gel. A NEB 1 kb extend ladder was run (lane 1). One replicate from each of the six extraction methods: Lucigen MasterPure Complete DNA and RNA Purification kit (MP, lane 2), Omega E.Z.N.A. Bacterial DNA kit (OMEGA, lane 3), Qiagen DNeasy Blood and Tissue (QIA, lane 4), GE Illustra Bacteria genomicPrep Mini Spin kit (GE, lane 5), Qiagen EZ1 DNA Tissue kit (EZ1, lane 6) and an in-house variation of Lucigen MasterPure Complete DNA and RNA Purification kit (MPIH, lane 7) developed at the National Microbiology Laboratory. The percentage of the lane that contains high-molecular-weight DNA (HMW; fragments ≥20 kb) is shown at the bottom of the gel.

DNA from each extraction method was run on a gel to examine the fragmentation. From the gel image, the proportion of the DNA ≥20 kb was determined using ImageJ [21]. There was significant variability in the fragmentation patterns observed for the different methods. Large amounts of HMW DNA were observed for MPIH and EZ1, with 97 and 91 % of the fragments ≥20 kb, respectively (Fig. 4). MP and OMEGA had large amounts of HMW DNA but also showed more shearing, which is reflected in their low HMW ratios of 62 and 66%, respectively (Fig. 4). QIA had overall a smaller quantity of HMW DNA compared to the other methods due to its lower concentration, but most of its DNA was HMW (87%) (Fig. 4). No HMW DNA was observed for GE; this is not surprising, as GE’s concentration was very low and HMW DNA may have been present but not observable (Fig. 4). All of the methods, with the exception of GE, produce sufficiently intact genomic DNA for ONT sequencing. MPIH, EZ1 and QIA are the most promising for producing long reads due to their higher ratios of HMW DNA.

Technical performance is only one aspect to consider when selecting an extraction method to implement within a routine diagnostic laboratory, so we also evaluated the consumable costs, hands-on time, total processing time and scalability for each of the kits. In terms of the cost of the kit per sample, QIA is the least expensive method (CAD $3.54) and EZ1 is the most expensive at CAD $11.25 (Table 1). When consumables costs are included, the difference in the cost per sample between the EZ1 method and the other extraction methods decreases (Table 1). Although, QIA and EZ1 are still the least and most expensive, the prices now range from CAD $5.99–12.32 (Table 1). In terms of total time for extraction (post-extraction purification is not included in these times), GE is the shortest (1 h 10 min), but it did not produce usable quantities of DNA (Table 1). All other methods required ~2 h, except for EZ1, which required just under 3.5 h, primarily due to our use of a 3 h incubation period for the lysis step. The manufacturer suggests that lysis for the EZ1 can be done in as little as 30 min, but this may impact on yields (Table 1). EZ1 requires the least amount of hands-on time (35 min); all other methods take between 1 and 2 h (Table 1). QIA offers the most flexibility of all of the methods in terms of throughput because both single-column and 96-well plate formats are available. The GE and OMEGA kits also use silica columns, but only offer single-column formats. Silica column technology is well established and a variety of automation solutions are available for it. EZ1 is already automated and, depending on the instrument, can process 6–24 samples in a run. Salt precipitation methods (MP and MPIH) are more challenging to scale up or automate due to the need to visualize the DNA pellet.

No single factor will determine which method is the best fit for all laboratories, so a combined score was generated for each extraction method (Table 2). The score was calculated by giving equal weight to the following factors: quantity, variability, purity, fragmentation, cost, hands-on time and total time (Table 2). Individual laboratories may wish to give different weights to different factors, but with equal weighting QIA, MPIH, and EZ1 were the top three performing methods in this study, representing one of each of the three different extraction technologies we examined in this study (Table 2).

### Sequencing/bioinformatics

**Fig. 5.**
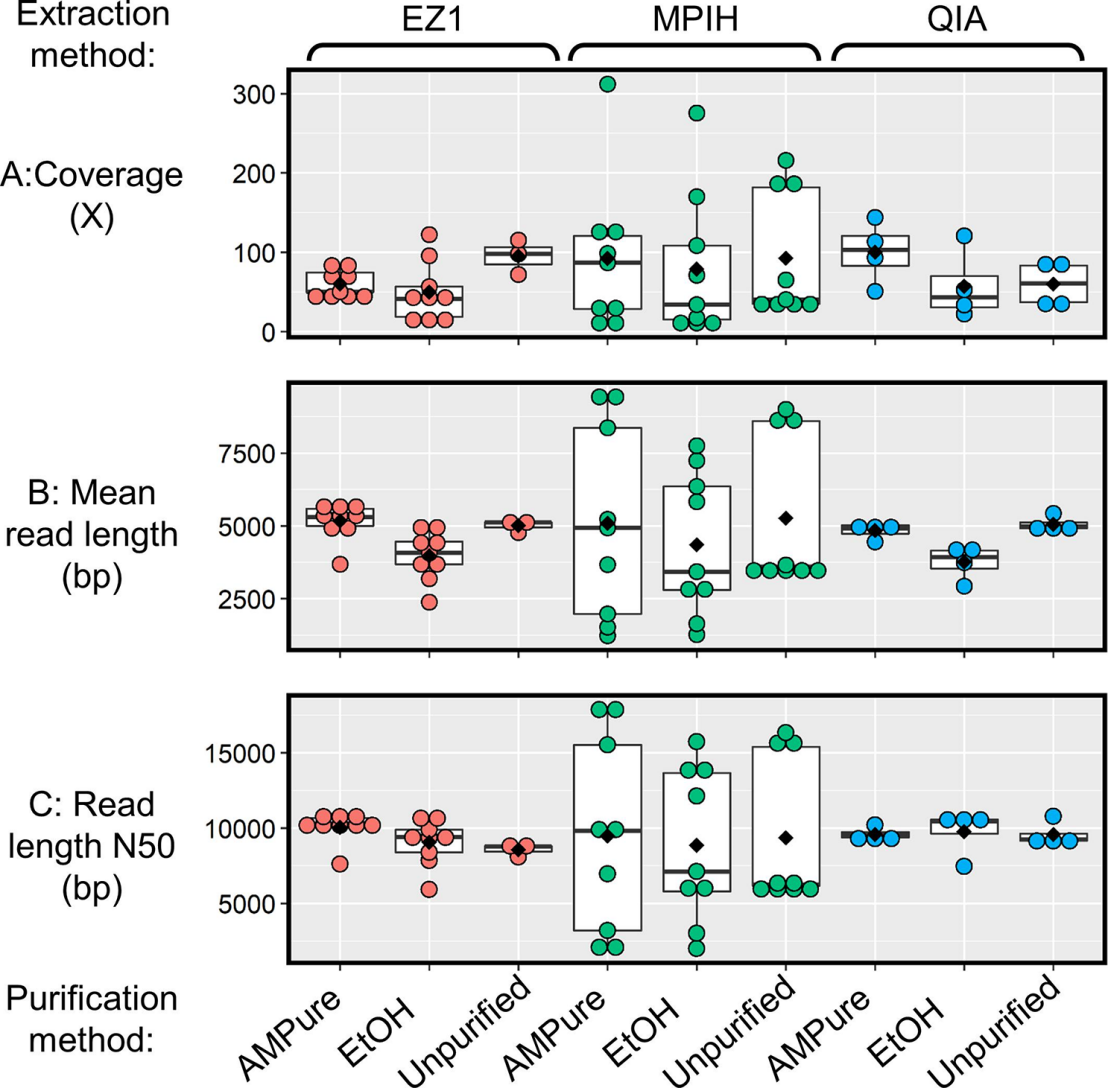
Box plots of (a) coverage, (b) mean read length and (c) read length N50 for each replicate grouped by the nine treatments. Each coloured dot is a replicate and replicates are coloured by extraction method: red, Qiagen EZ1 DNA Tissue kit (EZ1); green, an in-house variation of Lucigen MasterPure Complete DNA and RNA Purification kit (MPIH) developed at the National Microbiology Laboratory; and blue, Qiagen DNeasy Blood and Tissue kit (QIA). The purification methods are: ethanol precipitation (EtOH), Beckman Coulter AMPure XP Beads (AMPure), or none (unpurified). The treatments are organized on the *x*-axis first by extraction method and then by purification method. The black diamond is the mean for each treatment.

In general, for Illumina WGS of *Salmonella* in public health applications, a minimum of 30× coverage is targeted [28]. For ONT sequencing of *Salmonella *, Xian *et al.* found that 50× coverage was enough to capture over 99 % of SNPs and did not show significant differences in the number of allelic call errors for core genomics multilocus sequence typing (cgMLST) compared to 200× [29]. There was considerable variability in the sequencing depth across replicates, with coverage varying from 6 to 312× across replicates (Fig. 5). When grouped by extraction method, the mean coverage depth for all extraction methods was >60×, but MPIH had the highest mean sequencing depth with 88× (*n*=27) and EZ1 the lowest with 61× (*n*=21) (File S2). Post-extraction purification using either AMpure (81×) or EtOH (63×) had a negative effect on sequencing depth, with unpurified DNA having a depth of 85× (File S2). Sequencing metrics such as mean read length and read length N50 showed a similar pattern, where unpurified DNA performed the same or better than purified treatments (Fig. 5). Based on these results, there does not appear to be any benefit to AMpure or EtOH treatment of DNA prior to sequencing using ONT’s Rapid kit, which will save both time and money for laboratories.

None of the nine extraction–purification treatments were significantly different from each other based on the Kruskal–Wallis test with Bonferroni correction; however, there were large differences in variability between treatments. EZ1 and QIA replicates were very consistent in coverage (sd <45), mean read length (sd <900) and read length N50 (sd <1600), regardless of purification treatment, unlike MPIH, which showed high variability (sd >75, sd >2400 and sd >4800, respectively) across these metrics (Fig. 5). Although EZ1 and QIA were highly consistent for coverage, mean read length and read length N50, MPIH produced the highest values for each of these metrics, and in applications where this is necessary, there may be a benefit in using MPIH over EZ1 and QIA. The variability observed in MPIH treatments also reflects the high degree of variability observed for quantity with this method compared to the other two methods (Fig. 2). This is suggestive that high concentrations of HMW may not play as large a role in sequencing results as consistency in DNA fragment lengths; however, these results do not provide conclusive evidence that this is the case.

There are numerous factors that can cause variability between ONT sequencing runs (e.g. number of initial pores). Therefore, five sequencing runs were performed, each run with a separate library preparation on a different flow cell. By running each treatment on at least two difference sequencing runs, sequencing run variability could be factored into the analysis of the different treatments (File S3).

The reads generated from each replicate (*n*=60) were mapped to the reference genome. In each case, the mapped reads accounted for ≥98 % of the reference genome and for 28 replicates the mapped reads covered the reference completely. The worst two replicates were both extracted with MPIH and purified with EtOH; they were missing 63 and 13 kb compared to the reference genome. Therefore, regardless of the extraction or purification method used, genome recovery rate is very high, which means that any of these methods could be used to perform ONT WGS for diagnostic applications such as species identification and detection of virulence and AMR genes.

The goal of this paper is not to perform an exhaustive comparison of different *de novo* assembly methods (see Wick and Holt [9] for this analysis), but multiple assemblers and parameters were used on all 60 replicates to determine if any assembler was able to reconstruct a complete, circular genome from the reads. Similar to the findings of Wick and Holt [9], no single assembler performed best on all replicates. Of the two long read-only assemblers, Flye had the fewest replicates that did not generate a circular chromosome (11/60) compared to Canu (21/60) (File S2). The replicates with incomplete chromosomes were not always the same between assembly methods, which indicates that there is a strong need to evaluate multiple assembly approaches when assembling ONT sequence data (File S2). We included hybrid assemblers in the analysis to determine if hybrid approaches were better suited to generating circular chromosomes than long read-only. As expected, the hybrid assembly process only had six replicates (*n*=60), which did not produce a circular chromosome. Interestingly, Unicycler performed better when provided with the replicate’s long read assembly from Flye compared to the built-in long-read assembly using Miniasm (File S2).

In general, replicates that did not generate a circular chromosome with either long read assembler had a low total number of bases (<151 million bp, <31×), low mean read lengths (<2000 bp) and/or low read length N50s (<3200 bp) (File S2). There was considerable variability in the sequencing depth for replicates, so differences between the assembler results based on input coverage were examined. Both Flye and Canu showed a similar pattern where there was lower average coverage depth in the samples which failed to produce a complete chromosome compared to the successful replicates. Incomplete chromosomes for Flye and Canu had coverages of 23 and 39×, respectively, indicating that Flye is capable of closing genomes with lower sequencing input (File S2). Complete chromosomes for Flye and Canu had a much smaller difference in coverage with 87 and 95×, respectively (File S2). As expected, these results demonstrate that long read sequencing depth is definitely a factor in generating circular chromosomes but assembly methods differ in their coverage requirements. This is reinforced by the lack of a difference between complete and incomplete hybrid assemblies, where the average coverage for both was 75× (File S2). Given how the Unicycler algorithm prioritizes the Illumina assembly, it is not surprising that long read coverage was not a strong factor in generating a circular chromosome. There is no single best assembler for all datasets, but from our results for long read-only assemblers, we found Flye to be an excellent first choice for assembly of ONT data, with hybrid assembly with Unicycler using the Flye assembly outperforming the built-in Miniasm assembly.

When only using long read data for genome assembly, with either Flye or Canu, all QIA replicates (12/12) and most EZ1 replicates (20/21) generated reads that were sufficient to generate a circular chromosome (Table 3, File S2). Further, the one EZ1-EtOH replicate that was incomplete with both long read-only assemblers was circularized after incorporating short read data in a hybrid assembly method (Table 3). On the other hand, several replicates with MPIH-derived reads (8/27) had incomplete chromosomes in both of the long read-only assemblies (Flye and Canu) (Table 3). Interestingly, circular chromosomes were generated more frequently when MPIH DNA extracts were unpurified compared to when purified with either AMPure or EtOH (Table 3), suggesting that the additional handling during purification has a greater impact on MPIH than EZ1 or QIA. For MPIH, when incorporating short reads fewer replicates generated an incomplete chromosomes (5/27) compared to long read-only assemblers (8/27) (Table 3). However, five MPIH replicates did not generate complete chromosomes with any assembly method used, all of which were extracted with MPIH and purified with either AMPure or EtOH (Table 3).

**Table 3.** Summary of the number of replicates that were able to generate a complete chromosome with either Flye or Canu (long read data only) or any of the Unicycler parameter sets (short- and long read data) for the nine extraction–purification treatments. There are three extraction methods (EZ1, Qiagen EZ1 DNA Tissue kit; MPIH, in-house variation of Lucigen MasterPure Complete DNA and RNA Purification kit developed at the National Microbiology Laboratory; and QIA, Qiagen DNeasy Blood and Tissue kit) and three purification methods (AMPure, Beckman Coulter AMPure XP Beads; EtOH, ethanol precipitation; and unpurified, no purification)

Extraction and purification method		*n*	No. of replicates with complete chromosomes	
			Long read data only	Short and long read data
**EZ1**	**AMPure**	**9**	9	9
	**EtOH**	**9**	8	9
	**Unpurified**	**3**	3	3
**MPIH**	**AMPure**	**9**	6	6
	**EtOH**	**9**	5	7
	**Unpurified**	**9**	8	9
**QIA**	**AMPure**	**4**	4	4
	**EtOH**	**4**	4	4
	**Unpurified**	**4**	4	4
**TOTALS**		**60**	**51**	**55**

## Conclusion

All of the DNA extraction methods tested here with ONT sequencing resulted in very high recovery of the genome (reads mapped to ≥98 % of the reference). Therefore, any of these extraction methods could be used in diagnostic laboratory applications where reliable, appropriate reference genomes already exist. Important factors to consider when selecting an extraction method within a laboratory include: quantity, variability, purity, fragmentation, cost, hands-on time and total time. The importance of each of these factors for a specific laboratory and its applications may vary. Although reads from each replicate covered ≥98 % of the reference genome, this may not result in a circular genome, so for *de novo* assembly applications, more stringent method selection criteria may need to be applied based on the results we have described here.

We ranked Qiagen DNeasy Blood and Tissue kit (QIA) as the top DNA extraction method due to its low cost, consistency and short protocol length. It was also the only method where all replicates regardless of purification method resulted in a complete assembly with at least one assembler. Unpurified QIA extracts performed well in sequencing despite using lower amounts of DNA than recommended by ONT; therefore post-extraction concentration of the QIA DNA extracts is not required for the Rapid Sequencing kit. There were cases where ethanol precipitation (EtOH) and Beckman Coulter AMPure XP Beads (AMPure) post-extraction concentration resulted in worse sequencing results, so our general recommendation is to use these only if it is crucial due to very low concentrations or issues with purity. Since QIA generates small amounts of DNA (~20 ng/µl), it is not the best candidate if large amounts of DNA are required, such as in the case of the Ligation Sequencing kit. Although Qiagen EZ1 DNA Tissue kit (EZ1) performed similarly to QIA, it is much more expensive, does not offer a plate-based format and requires specialized equipment. However, EZ1 ranked highest in terms of having the shortest hands-on time requirements. To meet ONTs concentration requirements and prevent inhibition from the EZ1’s elution buffer, both QIA and EZ1 would require post-extraction purification; therefore, the cost and time increases due to post-extraction purification are the same for both methods. Overall, we expect our results to be applicable to other Enterobacteriaceae, but this will require separate evaluations to confirm. Our results should facilitate decision-making in diagnostic laboratories looking to implement ONT sequencing as a routine test.

## Peer review history

### VERSION 2

#### Editor recommendation and comments

https://doi.org/10.1099/acmi.0.000468.v2.1

© 2022 Schniete J. This is an open access peer review report distributed under the terms of the Creative Commons Attribution License.

**Jana Katharina Schniete**; Edge Hill University, Biology, St Helens Road, UNITED KINGDOM, Ormskirk

Date report received: 07 November 2022

Recommendation: Accept

**Comments**: Thank you for addressing all comments raised by the reviewers and myself.

#### Author response to reviewers to Version 1

Manuscript number: ACMI-D-22-00105

Title: Evaluation of five commercial DNA extraction kits using Salmonella as a model for

implementation of rapid Nanopore sequencing in routine diagnostic laboratories

Authors: Shannon H.C. Eagle; James Robertson; D. Patrick Bastedo; Kira Liu; John H.E. Nash

Dear Ms Eagle,

Thank you for submitting your paper to Access Microbiology. It has now been reviewed and I would like you to revise the paper in line with the reviewers' reports and any

Editorial Office requirements below. The reviewer reports can be found at the bottom of

the email.

Editor comments:

This is a study that would be of interest to the field and community.

The reviewers have highlighted minor concerns with the work presented. Please ensure that you address their comments.

In addition to the comments made by the reviewers, I would like to suggest to include a

diagram of the workflow with information what was evaluated at each step, this might help readers to follow the results better. Thank you for the suggestion. We have added a flow chart (Fig. 1). All subsequent figures were renumbered.

Please submit the revised version of your manuscript by 17/09/2022.

If you need longer than the suggested time-frame, please contact the Editorial Office in

advance to agree a different deadline at acmi@microbiologysociety.org.

Please note that some revisions are peer reviewed and submitting a revised paper does not guarantee that it will be accepted.

Editorial Office requirements:

1. Please upload figures as separate, high resolution, editable files. Acceptable file

types are PDF, GIF, TIFF, EPS, JPEG, PNG, SVG, and PPT. Please ensure the figures and legends are still in the main manuscript as the revised manuscript will be preprinted

again.

Submitting a revised paper:

To submit a revision, go to https://www.editorialmanager.com/acmi/ and log in as an

Author. You will see a menu item called ‘Submission Needing Revision’. You will find

your submission record there.

Kind regards,

Dr Jana Katharina Schniete

Editor, Access Microbiology

Microbiology Society| microbiologyresearch.org

Reviewers' comments and responses to custom questions:

Please rate the manuscript for methodological rigour

Reviewer 1: Good

Please rate the quality of the presentation and structure of the manuscript

Reviewer 1: Good

To what extent are the conclusions supported by the data?

Reviewer 1: Strongly support

Do you have any concerns of possible image manipulation, plagiarism or any other

unethical practices?

Reviewer 1: No:

If this manuscript involves human and/or animal work, have the subjects been treated in

an ethical manner and the authors complied with the appropriate guidelines?

Reviewer 1: Yes:

Reviewer 1 Comments to Author:

1.   Line 29: change "times" to "time"?

This has been corrected.

2. Line 292 please elaborate what is stopOnLowCoverage

In Canu, the default value for stopOnLowCoverage is 10. This means that a sample must have coverage greater than 10X for Canu to proceed with assembly. By lowering the value for stopOnLowCoverage, we were able to proceed with assembly for genomes with less than 10X coverage.

3. Line 311, if 77.8 ng/uL (as well as the following concentrations presented) is a mean of replicates, please present the standard deviation for each mean (or use any other values that can show the range or variation of the means)

The text has been updated to include standard deviations.

4. Line 372, does this conclusion take the post-extraction concentration time into

consideration? Since the yield of QIA is lower than required by ONT protocol, it for sure needs a post-extraction concentration step to increase the concentration of the DNA to ONT required threshold 54 ng/uL (400ng in total in 7uL). Please clarify it here for this statement. Although in the conclusion part the author claims that unpurified QIA extracts performed well in sequencing using lower amounts of DNA than recommended by ONT, users may want to pursue the required quantity 400ng most of the time to optimize the sequencing results.

Thank you for pointing out this point. We were not expecting the lower concentration unpurified DNA to outperform the concentrated DNA. Within the text we have made a few modifications. First, we clarified that the times listed here are for the extraction portion only. The conclusion was updated to address the increase in cost and time associated with post-extraction purification. Both QIA and EZ1 would likely require post-extraction purification to be concentrated enough; therefore, cost and time increases for post-extraction purification are equal for both methods.

5. Line 389, the paper Subtyping Evaluation of Salmonella Enteritidis Using Single

Nucleotide Polymorphism and Core Genome Multilocus Sequence Typing with Nanopore Reads | Applied and Environmental Microbiology (asm.org)

(https://journals.asm.org/doi/10.1128/aem.00785-22) can be of help for the recommended sequencing depth of genome coverage for WGS with ONT data.

Thank you for brining this paper to our attention. We have updated the text to include the findings from Zian *et al.*, 2022 concerning sequencing depth recommendations for SNP and cgMLST analysis of *Salmonella*using ONT data.

6. Line 392, the data yield or sequencing depth of each flow cell is dependent on several factors including input DNA quantity, length of each DNA read, number of DNA reads (can be calculated using input DNA quantity and N50 DNA length of all reads), initial number of active pores, please specify or discuss the potential influence of these factors to the final data yield along with the comparison between methods, otherwise, the comparison can be bias.

This is definitely a valuable piece of information to include in the manuscript. We have added a paragraph to the main text (line 422) and a Supplemental File (Supplemental File 3). Numerous factors influence ONT sequencing results. Within our experiments, we controlled for variability in sequencing performance as best as possible by using multiple independent library preparations and sequencing runs. Since this may also be a concern or of interest to readers, Supplemental File 3 shows the Figure 4 (now Figure 5) box plot with replicates coloured by sequencing run. This figure allows readers to see the variability between different sequencing runs.

7. Please elaborate on this "Although ≥ 98% of the genome is represented by reads", what does "represented by reads" indicate?

We mapped the reads from each replicate to a PacBio reference genome for this isolate and determined all of the regions of the reference genome with ≥ 1X sequence depth which sum to 98%. We have updated the manuscript to clarify this point.

Please rate the manuscript for methodological rigour

Reviewer 2: Very good

Please rate the quality of the presentation and structure of the manuscript

Reviewer 2: Good

To what extent are the conclusions supported by the data?

Reviewer 2: Strongly support

Do you have any concerns of possible image manipulation, plagiarism or any other

unethical practices?

Reviewer 2: No:

If this manuscript involves human and/or animal work, have the subjects been treated in

an ethical manner and the authors complied with the appropriate guidelines?

Reviewer 2: Yes:

Reviewer 2 Comments to Author:

The manuscript presented evaluates various DNA extraction methods for use with Nanopore sequencing using the Rapid kit with a specific view as to how useful each is in the context of a diagnostic laboratory. A Salmonella enterica strain which has previously been sequenced to completion with high-quality combinatorial assembly using both Illumina and PacBio sequencing chemistry. Five different commercial

extraction kits were tested here, spanning different approaches to DNA extraction, in

addition, an altered method for one of these kits was trailed separately. The manuscript

is generally well presented, and the results seem to be properly interpreted. All raw

data in the form of sequencing reads are available online under bio-project: PRJNA768992. Each kit is evaluated in terms of the quality and quantity of the DNA extracted as well as the resulting sequence data obtained, from this it is concluded that the Qiagen DNeasy Blood and Tissue kit is the most suitable.

1. Methodological rigour

The methods for extraction of DNA as well as the evaluation of the different kits have

been conducted toughly. The use of a scoring system to account for the various factors is a sensible method of comparison. The only methodological improvement I could suggest would be a statistical analysis of the data shown in figure 4 - I couldn't see a mention of this having been done but it would be relevant to indicate whether the differences in sequence output between extraction kits were significant.

Due to the visual overlap of the samples, we did not originally perform a statistical analysis due to the assumption that there would not be a significant difference in the means. Thank you for pointing out this omission. We performed Kruskal-Wallis test with Bonferroni correction and this confirmed that none of the means for the treatments were significantly different. We calculated standard deviation for each of the nine treatments and think that this strengthens the results. We have updated the text to reflect this analysis.

In addition, I would suggest that some sort of evaluation of the accuracy of the

assemblies obtained would be relevant but not necessary to this work. If the analysis has been done already, the inclusion of information such as the number of SNPs detected in each assembly compared to the reference would be of interest since, as was mentioned, high coverage is often required to account for the higher error rate with Nanopore sequencing.

We agree that there is definitely value in examining the resulting assemblies. However, this is a complex analysis to do thoroughly and we believe that limiting the analysis to just having enough data for different assemblers to close the genome be an appropriate stopping point for our goal of comparing the sequencing results obtained from different extraction methods.

2. Presentation of results

The results are generally well presented with DNA quantification, purity and fragment

length all reported as well as the coverage, read length and N50 values for each

extraction method. If possible, I believe two small changes could be made to the figures

to improve readability. These are

1.   Figure 1 included the average total DNA for each

extraction method The average has been added to Figure 1 (now Figure 2).

2.   Table 3 showed "the percentage of replicates with complete chromosomes" instead of "number with incomplete chromosomes". Table 3 has been changed from incomplete chromosomes to complete chromosomes.

There is potentially some confusion with the references to supplementary data in the manuscript. For example, data from supplementary file S2 is referred to as being both S1 and S2. Thank you for pointing this out. We have gone through and changed all Data S# to Supplementary File #. There was a figure at the end of Supplementary File 1 that showed the breakdown of replicates and whether or not a circular chromosome was generated. However, this information is also found in Supplementary File 2; therefore, we removed this Figure.

Below I have outlined what I found to be the associations as best I could.

Line 210: Data S1 - Manufacturers' protocols with modifications are described

Is "Supplemental File 1"

This has been corrected.

Lines 426, 429, 457: Data S1 - Assembly methods that generate circular chromosome

Is "Supplementary File 2"

This has been corrected.

Line 282: Data S2 - file containing accession numbers

Is "Supplementary File 2"

This has been corrected.

Lines 395, 397: Data S2 mean coverage grouped by extraction method

I was unable to find the data this refers to

The means have been added to Supplemental File 2.

Line 437: Data S2 mean read length and N50s

Is "Supplementary File 2"

This has been corrected.

Lines 443, 445 and 449: Data S2 coverage for data that produced incomplete chromosomes for Flye and Canu assemblies

I was unable to find the data this refers to - figures are similar to what would be obtained by averaging the data given in "Supplementary File 2" but not quite the same as my calculations.

Thank you for bringing this to our attention. This data calculation has been added to Supplemental File 2 and the reference within the manuscript has been updated to address potential confusion. There are several coverage estimates in Supplementary File 2; the estimates used for this calculation are covTotalBases which is calculated from TotalBases and the reference genome size. We have confirmed that the numbers in the manuscript are correct.

Supplemental Figure 1 - I didn't find a reference to this figure, and it was lacking a

legend.

Thank you for highlighting this issue, we have removed this Figure from the Supplemental File.

3. How the style and organization of the paper communicates and represents key findings

The key finding here is that the Qiagen DNeasy Blood and Tissue Kit without any postextraction concentration is the best extraction method for Salmonella in a diagnostic

laboratory setting. This is communicated clearly with caveats such as cost, hands-on time and batch size accounted for.

4. Literature analysis or discussion

Due to the nature of this manuscript, the scope for analysis is limited, however, the

relevant literature and explanation of the use and limitations of sequencing in a

diagnostic setting are included. The results are appropriately evaluated and discussed in the context.

5. Any other relevant comments

In general, I thought that this manuscript achieved the intended outcome of evaluating

various extraction methods and that this will be relevant for diagnostic testing of

Salmonella and probably more widely. This type of result is of value as it will allow

those with fewer resources or time to make more informed decisions on methods. Though it may be possible for the findings here to be communicated in a slightly more concise way.

For this type of article, a more succinct methods and results section, in particular,

would increase readability.

Typos -

Line 40 "WGS" should be defined for the first use This has been corrected.

Line 158 carbapenam should be carbapenem This has been corrected

Line 465 an incomplete assembly? Thank you for bringing this to our attention. We have reviewed this section and confirmed that in this sentence we are specifically talking about the chromosome.

### VERSION 1

#### Editor recommendation and comments

https://doi.org/10.1099/acmi.0.000468.v1.5

© 2022 Schniete J. This is an open access peer review report distributed under the terms of the Creative Commons Attribution License.

**Jana Katharina Schniete**; Edge Hill University, Biology, St Helens Road, UNITED KINGDOM, Ormskirk

Date report received: 18 August 2022

Recommendation: Minor Amendment

**Comments**: This is a study that would be of interest to the field and community. The reviewers have highlighted minor concerns with the work presented. Please ensure that you address their comments. In addition to the comments made by the reviewers, I would like to suggest to include a diagram of the workflow with information what was evaluated at each step, this might help readers to follow the results better.

#### Reviewer 2 recommendation and comments

https://doi.org/10.1099/acmi.0.000468.v1.3

© 2022 Beaton A. This is an open access peer review report distributed under the terms of the Creative Commons Attribution License.

**Ainsley Beaton**; John Innes Centre, John Innes Centre, Colney Lane, Norwich, UNITED KINGDOM

https://orcid.org/0000-0002-3799-2600

Date report received: 10 August 2022

Recommendation: Minor Amendment

**Comments**: The manuscript presented evaluates various DNA extraction methods for use with Nanopore sequencing using the Rapid kit with a specific view as to how useful each is in the context of a diagnostic laboratory. A Salmonella enterica strain which has previously been sequenced to completion with high-quality combinatorial assembly using both Illumina and PacBio sequencing chemistry. Five different commercial extraction kits were tested here, spanning different approaches to DNA extraction, in addition, an altered method for one of these kits was trailed separately. The manuscript is generally well presented, and the results seem to be properly interpreted. All raw data in the form of sequencing reads are available online under bio-project: PRJNA768992. Each kit is evaluated in terms of the quality and quantity of the DNA extracted as well as the resulting sequence data obtained, from this it is concluded that the Qiagen DNeasy Blood and Tissue kit is the most suitable. Methodological rigour The methods for extraction of DNA as well as the evaluation of the different kits have been conducted toughly. The use of a scoring system to account for the various factors is a sensible method of comparison. The only methodological improvement I could suggest would be a statistical analysis of the data shown in figure 4 - I couldn't see a mention of this having been done but it would be relevant to indicate whether the differences in sequence output between extraction kits were significant. In addition, I would suggest that some sort of evaluation of the accuracy of the assemblies obtained would be relevant but not necessary to this work. If the analysis has been done already, the inclusion of information such as the number of SNPs detected in each assembly compared to the reference would be of interest since, as was mentioned, high coverage is often required to account for the higher error rate with Nanopore sequencing. 2. Presentation of results The results are generally well presented with DNA quantification, purity and fragment length all reported as well as the coverage, read length and N50 values for each extraction method. If possible, I believe two small changes could be made to the figures to improve readability. These are 1. Figure 1 included the average total DNA for each extraction method and 2. Table 3 showed "the percentage of replicates with complete chromosomes" instead of "number with incomplete chromosomes". There is potentially some confusion with the references to supplementary data in the manuscript. For example, data from supplementary file S2 is referred to as being both S1 and S2. Below I have outlined what I found to be the associations as best I could. Line 210: Data S1 - Manufacturers' protocols with modifications are described Is "Supplemental File 1" Lines 426, 429, 457: Data S1 - Assembly methods that generate circular chromosome Is "Supplementary File 2" Line 282: Data S2 - file containing accession numbers Is "Supplementary File 2" Lines 395, 397: Data S2 mean coverage grouped by extraction method I was unable to find the data this refers to Line 437: Data S2 mean read length and N50s Is "Supplementary File 2" Lines 443, 445 and 449: Data S2 coverage for data that produced incomplete chromosomes for Flye and Canu assemblies I was unable to find the data this refers to - figures are similar to what would be obtained by averaging the data given in "Supplementary File 2" but not quite the same as my calculations. Supplemental Figure 1 - I didn't find a reference to this figure, and it was lacking a legend. 3. How the style and organization of the paper communicates and represents key findings The key finding here is that the Qiagen DNeasy Blood and Tissue Kit without any post-extraction concentration is the best extraction method for Salmonella in a diagnostic laboratory setting. This is communicated clearly with caveats such as cost, hands-on time and batch size accounted for. 4. Literature analysis or discussion Due to the nature of this manuscript, the scope for analysis is limited, however, the relevant literature and explanation of the use and limitations of sequencing in a diagnostic setting are included. The results are appropriately evaluated and discussed in the context. 5. Any other relevant comments In general, I thought that this manuscript achieved the intended outcome of evaluating various extraction methods and that this will be relevant for diagnostic testing of Salmonella and probably more widely. This type of result is of value as it will allow those with fewer resources or time to make more informed decisions on methods. Though it may be possible for the findings here to be communicated in a slightly more concise way. For this type of article, a more succinct methods and results section, in particular, would increase readability. Typos - Line 40 "WGS" should be defined for the first use Line 158 carbapenam should be carbapenem Line 465 an incomplete assembly?

*Please rate the manuscript for methodological rigour*

Very good

*Please rate the quality of the presentation and structure of the manuscript*

Good

*To what extent are the conclusions supported by the data?*

Strongly support

*Do you have any concerns of possible image manipulation, plagiarism or any other unethical practices?*

No

*Is there a potential financial or other conflict of interest between yourself and the author(s)?*

No

*If this manuscript involves human and/or animal work, have the subjects been treated in an ethical manner and the authors complied with the appropriate guidelines?*

Yes

#### Reviewer 1 recommendation and comments

https://doi.org/10.1099/acmi.0.000468.v1.4

© 2022 Anonymous. This is an open access peer review report distributed under the terms of the Creative Commons Attribution License.

**Anonymous.**

Date report received: 14 July 2022

Recommendation: Minor Amendment

**Comments**: 1. Line 29: change "times" to "time"? 2. Line 292 please elaborate what is stopOnLowCoverage 3. Line 311, if 77.8 ng/uL (as well as the following concentrations presented) is a mean of replicates, please present the standard deviation for each mean (or use any other values that can show the range or variation of the means) 4. Line 372, does this conclusion take the post-extraction concentration time into consideration? Since the yield of QIA is lower than required by ONT protocol, it for sure needs a post-extraction concentration step to increase the concentration of the DNA to ONT required threshold 54 ng/uL (400ng in total in 7uL). Please clarify it here for this statement. Although in the conclusion part the author claims that unpurified QIA extracts performed well in sequencing using lower amounts of DNA than recommended by ONT, users may want to pursue the required quantity 400ng most of the time to optimize the sequencing results. 5. Line 389, the paper Subtyping Evaluation of Salmonella Enteritidis Using Single Nucleotide Polymorphism and Core Genome Multilocus Sequence Typing with Nanopore Reads | Applied and Environmental Microbiology (asm.org) (https://journals.asm.org/doi/10.1128/aem.00785-22) can be of help for the recommended sequencing depth of genome coverage for WGS with ONT data. 6. Line 392, the data yield or sequencing depth of each flow cell is dependent on several factors including input DNA quantity, length of each DNA read, number of DNA reads (can be calculated using input DNA quantity and N50 DNA length of all reads), initial number of active pores, please specify or discuss the potential influence of these factors to the final data yield along with the comparison between methods, otherwise, the comparison can be bias. 7. Please elaborate on this "Although ≥ 98% of the genome is represented by reads", what does "represented by reads" indicate?

*Please rate the manuscript for methodological rigour*

Good

*Please rate the quality of the presentation and structure of the manuscript*

Good

*To what extent are the conclusions supported by the data?*

Strongly support

*Do you have any concerns of possible image manipulation, plagiarism or any other unethical practices?*

No

*Is there a potential financial or other conflict of interest between yourself and the author(s)?*

No

*If this manuscript involves human and/or animal work, have the subjects been treated in an ethical manner and the authors complied with the appropriate guidelines?*

Yes

#### SciScore report

https://doi.org/10.1099/acmi.0.000468.v1.1

© 2022 The Authors. This is an open-access article report distributed under the terms of the Creative Commons License.

#### iThenticate report

https://doi.org/10.1099/acmi.0.000468.v1.2

© 2022 The Authors. This is an open-access article report distributed under the terms of the Creative Commons License.

## Supplementary Data

Supplementary material 1

Supplementary material 2

Supplementary material 3
