# Supplementary material for: Evaluation of five commercial DNA extraction kits using Salmonella as a model for implementation of rapid Nanopore sequencing in routine diagnostic laboratories
Source: Access Microbiol. 2023 Feb 21;5(2):000468.v3. doi: 10.1099/acmi.0.000468.v3 (PMC9996181; doi:10.1099/acmi.0.000468.v3)
Supplement: Supplementary material 1 [file acmi-5-468.v3-s001.pdf]

Supplemental File 1

Supplemental Methods

Extractions

The Lucigen MasterPure Complete Total Nucleic Acid Purification (MP) followed the DNA Purification Cell Samples protocol. The following modifications were made: samples were cooled to 37°C after incubating at 65°C, samples were air-dried after the removal of residual ethanol, and samples were eluted with 100 µL of molecular grade water instead of 35 µL of TE Buffer.

The In-House version of the Lucigen MasterPure Complete Total Nucleic Acid Purification (MPIH) differed from the manufacturer’s DNA Purification Cell Samples protocol. The 2X version of the Tissue and Cell Lysis buffer was used instead of the 1X version, and 3 µL of proteinase K was added to each sample instead of 1 µL. The 65°C incubation was done on a thermomixer shaking at 300 rpm, instead of vortexing every 5 minutes. Samples were cooled to room temperature before incubating at 4°C for 10 minutes. An additional 200 µL of MPC Protein Precipitate Reagent (MP = 150 µL, MPIH = 350 µL) was added. An additional 3 µL RNaseA (MP = 1 µL, MPIH = 4 µL) was added, and the RNaseA incubation time was reduced to 5 minutes from 30 minutes. Ice incubation was not done following the RNaseA treatment. An additional 100 µL of Isopropanol (MP = 500 µL, MPIH = 600 µL) was added. During the wash steps, all centrifugations were done at 14,000 x *g*, and supernatants were removed using a pipette. Only a single ethanol wash was done; however, the sample was centrifuged before removing the ethanol wash (10 minutes at 14,000 x *g* at 4°C). After the addition of 100 µL of molecular grade water, samples were incubated overnight at room temperature to resuspend the DNA.

The Omega E.Z.N.A. Bacterial DNA Kit (OMEGA) protocol was modified as follows: cells were lysed at 55°C for 1 hour and DNA was eluted with 100 µL of molecular grade water instead of 50 to 100 µL of the kit’s elution buffer.

The Qiagen DNeasy Blood and Tissue Kit (QIA) followed the Purification of Total DNA from Animal Tissues Spin-Column Protocol DNA with a Pre-Treatment for Gram-Negative Bacteria (using a 1 hour lysis). DNA was eluted with 100 µL of molecular grade water instead of 200 µL of the kit’s elution buffer.

The GE Illustra Bacterial Genomic Prep Mini Spin Kit (GE) followed the Gram-negative bacteria protocol recommended by the manufacturer. The only modification was eluting the DNA with 100 μL of molecular grade water instead of 200 μL of the kit's elution buffer.

The Qiagen EZ1 DNA Tissue Kit (EZ1) used the Qiagen Advanced or EZ1 Advanced XL automated instruments and eluted in 100 μL of elution buffer. The following modifications were used: cultures were spun down at 4000 x *g* for 10 minutes, pellets were resuspended in 190 μL of Buffer G2 and 10 μL of proteinase K, and samples were incubated at 59°C for 0.5 to 4 hours.

Extraction and Purification of Sequencing Batches

Prior to sequencing, two new batches of DNA extracts (batch one and two) were extracted and purified. Three different extraction methods (EZ1, MPIH, and QIA) were subjected to one of two purification methods (EtOH and AMPure), or alternatively were left Unpurified. Twenty 1 mL aliquots of overnight culture were processed in parallel for each extraction method.

Each of the 20 replicates for each of the three extraction method were eluted in 50 μL of molecular grade water (QIA and MPIH) or elution buffer (EZ1). Samples were quantified with the Qubit Broad Range Quantification Kit (Invitrogen) as per manufacturer's protocol. For each extraction method, the fourteen samples with the highest concentrations were pooled. From this pool, six 80 μL aliquots were made per extraction method, three for each of the two purification method. Samples were purified as described in the main text.

Extraction batch one was used for sequencing runs one and two, and extraction batch two was used for sequencing runs three, four, and five. In three cases, EZ1-EtOH (extraction batch one), MPIH-AMPure (extraction batch two), and QIA-EtOH (extraction batch two), one of the three purified replicates did not have enough DNA post-purification; therefore, in each of these cases one of the two replicates with enough DNA was sequenced twice.
