# Supplementary material for: Evaluation of five commercial DNA extraction kits using Salmonella as a model for implementation of rapid Nanopore sequencing in routine diagnostic laboratories
Source: Access Microbiol. 2023 Feb 21;5(2):000468.v3. doi: 10.1099/acmi.0.000468.v3 (PMC9996181; doi:10.1099/acmi.0.000468.v3)
Supplement: Supplementary material 3 [file acmi-5-468.v3-s003.pdf]

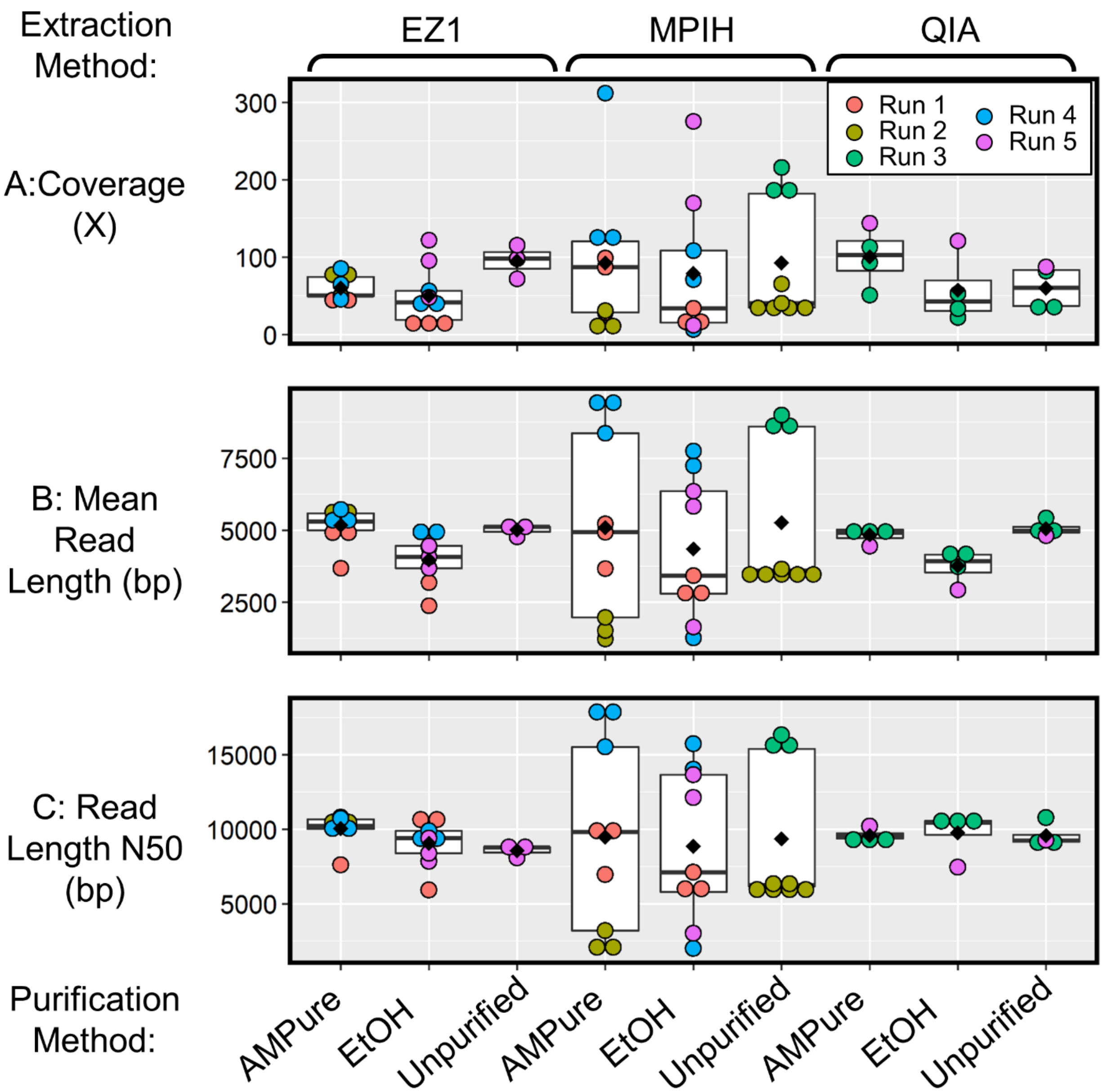

Supplemental File 3: Box plots of (A) coverage, (B) mean read length, and (C) read length N50 for each replicate grouped by the nine treatments. Each coloured dot is a replicate and replicates are coloured by sequencing run: red, run 1; yellow, run 2; green, run 3; blue, run 4; and pink, run 5. The extraction methods are: Qiagen EZ1 DNA Tissue Kit (EZ1); an in-house variation of Lucigen MasterPure Complete DNA and RNA Purification Kit (MPIH) developed at the National Microbiology Laboratory; and Qiagen DNeasy Blood and Tissue Kit (QIA). The purification methods are: ethanol precipitation (EtOH); Beckman Coulter AMPure XP Beads (AMPure); or none (Unpurified). The treatments are organized on the x-axis first by extraction method then by purification method. The black diamond is the mean for each treatment.
